# A proteomics approach to identify targets of the ubiquitin-like molecule Urm1 in *Drosophila melanogaster*

**DOI:** 10.1371/journal.pone.0185611

**Published:** 2017-09-27

**Authors:** Behzad Khoshnood, Ingrid Dacklin, Caroline Grabbe

**Affiliations:** Department of Molecular Biology, Umeå University, Umeå, Sweden; Biomedical Sciences Research Center Alexander Fleming, GREECE

## Abstract

By covalently conjugating to target proteins, ubiquitin-like modifiers (UBLs) act as important regulators of target protein localization and activity, thereby playing a critical role in the orchestration of cellular biology. The most ancient and one of the least studied UBLs is Urm1, a dual-function protein that in parallel to performing similar functions as its prokaryotic ancestors in tRNA modification, also has adopted the capacity to conjugate to cellular proteins analogous to ubiquitin and other UBL modifiers. In order to increase the understanding of Urm1 and its role in multicellular organisms, we have used affinity purification followed by mass spectrometry to identify putative targets of Urm1 conjugation (urmylation) at three developmental stages of the *Drosophila melanogaster* lifecycle. Altogether we have recovered 79 Urm1-interacting proteins in *Drosophila*, which include the already established Urm1 binding partners Prx5 and Uba4, together with 77 candidate urmylation targets that are completely novel in the fly. Among these, the majority was exclusively identified during either embryogenesis, larval stages or in adult flies. We further present biochemical evidence that four of these proteins are covalently conjugated by Urm1, whereas the fifth verified Urm1-binding protein appears to interact with Urm1 via non-covalent means. Besides recapitulating the previously established roles of Urm1 in tRNA modification and during oxidative stress, functional clustering of the newly identified Urm1-associated proteins further positions Urm1 in protein networks that control other types of cellular stress, such as immunological threats and DNA damage. In addition, the functional characteristics of several of the candidate targets strongly match the phenotypes displayed by *Urm1*^*n123*^ null animals, including embryonic lethality, reduced fertility and shortened lifespan. In conclusion, this identification of candidate targets of urmylation significantly increases the knowledge of Urm1 and presents an excellent starting point for unravelling the role of Urm1 in the context of a complex living organism.

## Introduction

Posttranslational modification of proteins by ubiquitin and the related ubiquitin-like proteins (UBLs) has repeatedly been demonstrated as a key strategy of eukaryotic cells to regulate most aspects of cellular life. The growing family of UBLs share two common features, a β-grasp globular superfold and C-terminal Glycine-Glycine (GG) motif, used for conjugation to target proteins. At present, the UBL family includes ubiquitin, Smt3 (Sumo-1, -2, -3), NEDD8, FUB1, FAT10, ISG15, Atg12, Atg8, UFM1 and Urm1 [1–3]. Initially, the research on UBLs was strongly focused on ubiquitylation, but in the past decades essential functions have also been ascribed to other UBLs, including Atg12 in autophagy and Sumo in multiple nuclear processes [3]. The role of UBLs as molecular switches, together with their high degree of evolutionary conservation, strongly emphasizes the need for further characterization also of other UBLs, several of which are still poorly understood.

Similar to ubiquitin, all UBLs conjugate to their target proteins through the conserved GG-motif, an event that in the case of ubiquitin and the Sumo proteins occur via a canonical enzymatic pathway in which UBL-specific E1 activating, E2 conjugating and E3 ligating enzymes are sequentially activated [1–3]. The other UBLs appear to follow non-canonical modes of activation, where unique combinations of one or a few E1, E2 and/or E3 enzymes are utilized for conjugation [1]. Whereas most UBLs appear to conjugate to their target proteins as single moieties, ubiquitin and SUMO additionally have the capacity to form chains of different lengths, linkages and blends, generating an extensive variation in the UBL modification landscape [4]. The number of targets varies extensively between different UBLs, ranging from potentially thousands for ubiquitin and SUMO to a relatively small number for Atg12 [1].

The evolutionary origin of posttranslational modification by UBLs is believed to have started with the acquisition of dual functions in the most ancient UBL, the Ubiquitin-related modifier 1 (Urm1). Initially, Urm1 was pinpointed as a UBL in *S*. *cerevisiae*, based on its sequence similarities with the prokaryotic sulfur carrier proteins MoaD (the small subunit of molybdopterin (MPT) synthase) and ThiS, involved in the biosynthesis of molybdopterin and thiamine, respectively [5]. At this point Urm1 was characterized as a protein modifier, which in concert with its E1 activating enzyme Uba4 could covalently conjugate to multiple target proteins in yeast [5], but further studies showed that Urm1 in addition had maintained a sulfur transfer activity, similar to its prokaryotic ancestors [6–10]. Specifically, Urm1 was later found to play essential roles as a sulfur carrier in the thio-modification of lysine (UUU), glutamine (UUG) and glutamate (UUC) tRNAs [6–10]. Together, these observations lead to the conclusion that through evolution Urm1 had complemented its sulfur carrier activity with the ability to conjugate to target proteins, and that Urm1 hence represents the evolutionary link between ancient UBL progenitors and the protein conjugation systems used by eukaryotes [10, 11]. The link between prokaryotic sulfur carriers and eukaryotic UBLs is today well established, not least by the structural investigations demonstrating common features both in their β-grasp topology and in the molecular mechanisms used for sulfur transfer versus protein conjugation [12–16].

Conjugation of Urm1 to its target proteins, known as urmylation, is highly conserved through evolution and shows strong resemblance to other eukaryotic UBLs, such as the utilisation of a UBL-specific E1 activating enzyme (Uba4/MOCS3) and the covalent conjugation to lysine residues in the target proteins [5, 17–19]. However, in contrast to other eukaryotic UBLs, the initial activation of Urm1 by Uba4, during which Uba4 adenylates and transfers sulfur to the C-terminus of Urm1, prepares Urm1 for protein conjugation as well as for tRNA modification. In this way, the dual functions of Urm1 are chemically linked since the resulting thiocarboxylated Urm1 can be recognized by both Ncs2/Ncs6 to mediate tRNA thiolation, and/or the cellular machinery that induces protein urmylation [8, 9, 19–21]. In agreement, tRNA modification and protein urmylation in yeast are both dependent on sulfur supply as well as proteins involved in Urm1 activation [22]. In this aspect, it is highly interesting to note that in multiple species of archaea, which phylogenetically are placed closer to eukaryotes than prokaryotes, there are also UBL proteins that share the dual ability to function in tRNA thiolation and protein conjugation [23–26]. Providing further connections to eukaryotic UBL systems, these include the UBLs SAMP1 and SAMP2 in *H*. *volcanii*, of which SAMP2 similar to ubiquitin can form chains [24], as well as Urm1 in *S*. *acidocaldarius* that utilizes urmylation to induce proteasomal degradation of its targets [23]. However, this appearance of dual functions of UBL proteins in sulfur transfer and protein conjugation in archaea was recently challenged by the discovery that the UBL TtuB in prokaryotic *T*. *thermophilus* in addition to act as a sulfur carrier in tRNA modification, also displays the capacity to conjugate to target proteins [16, 27].

Whereas the role of Urm1 as a sulfur carrier in the thiomodification of tRNAs has been described in detail by several independent groups [6–10, 28], its role as a target specific protein modifier is still poorly understood. Functionally, Urm1 was initially described as important for budding and growth at high temperatures in yeast [5, 18], as well as cytokinesis in HeLa cells [10], but has since then primarily been associated with the cellular defence against oxidative stress [17–19]. A role of Urm1 in oxidative stress has strongly been supported by the observation of increased levels of urmylation and altered sensitivity during oxidative stress in multiple model systems, including yeast, mammalian cells and in *D*. *melanogaster* [17, 19, 29]. However, the attempts to identify Urm1 targets and understand the biological role of target protein urmylation during these processes have been sparse. For a long time, the only verified target of Urm1 conjugation was the Alkyl hydroxide reductase I (Ahp1), identified in *S*. *cerevisiae* by Goehring and co-workers [17], but the list of targets was significantly extended by a proteomics-based approach used by Van der Veen et al, pinpointing 21 novel targets in mammalian cells [19], as well as a similar study performed by Humbard et al. that defined Urm1 targets in *H*. *volcanii* [24]. Other groups, including ourselves, have further verified and studied already established Urm1 targets [29, 30], including our confirmation of Urm1 conjugation to Prx5, the *Drosophila* orthologue of Ahp1 [29]. Even though urmylation appears to be critical for the response against oxidative stress in multiple species [17, 19, 29], the phenotypes displayed by *Urm1* null fly mutants suggest that Urm1 also has other functions during different stages of fly development ([29]). However, the targets and molecular pathways affected by urmylation in these processes are currently unknown.

In this study, we have for the first time investigated the urmylation landscape in the higher complexity of a multicellular organism and in different developmental contexts. By utilizing a combination of *Drosophila* genetics, proteomics and bioinformatics, we here report the identification of 79 Urm1-associated proteins, of which we have verified four to be covalently conjugated by Urm1 and one as a non-covalent binding partner of Urm1. We further report a highly divergent pattern of urmylation during three key developmental stages of the fly life cycle, including embryogenesis, late larval development and adulthood, placing Urm1 in protein networks that regulate several distinct biological processes.

## Materials and methods

### Fly strains

Standard *Drosophila* husbandry procedures were employed. Flies were raised and crossed at room temperature (RT) unless otherwise stated. *white*^*1118*^ was used as wild type control. *Actin5C-GAL4* and *GFP-TRAP*:*GILT1* (y1 w*; P{PTT-GC}GILT1^*CC00817*^) was obtained from Bloomington *Drosophila* Stock Center (BDSC), Indiana, USA. *UAS*:*HA-Ciao1* (*M{UAS-Ciao1*.*ORF*.*3xHA*.*GW}ZH-86Fb}*, stock F002753) and *UAS*:*HA-MsrA* (*M{UAS-Eip71CD*.*ORF*.*3xHA}*, stock F001070) was obtained from the FlyORF library (Zurich ORFeome Project) [31]. *pCasper4*:*Crammer-GFP* transgenic flies were a kind gift from Prof. T. Preat [32]. *Urm1*^*n123*^ and *UAS*:*Flag-Urm1*^*WT*^ has been described previously [29] and was used to generate the *UAS*:*Urm1*^*3xFlag*^ rescued *Urm1*^*n123*^ strain *Actin5C-GAL4/UAS*:*Flag-Urm1*^*WT*^*;Urm1*^*n123*^*/Urm1*^*n123*^ through established *Drosophila* genetic crossing schemes.

### Immunoprecipitation and Western blot

#### Protein lysate preparation

*Drosophila* embryos, larvae or flies were homogenized in Lysis buffer A (50 mM HEPES pH 7.4, 150 mM NaCl, 1 mM EDTA, 1 mM EGTA, 1% Triton X-100, 10% Glycerol, 25 mM NAF, 10 μM ZnCl_2_), at all times supplemented with Complete Protease Inhibitor Cocktail (Roche), 1 mM PMSF and 10 mM N-Ethylmaleimide (Sigma-Aldrich). The lysates were cleared by centrifugation at top speed for 15 min at 4°C and the total protein concentration was determined using standard Bradford protein assay (Bio-Rad). 1–2 mg of total protein were used for immunoprecipitation, unless otherwise stated.

#### Immunoprecipitation with FlagM2 magnetic beads

Immunoprecipitations were performed using Anti-FLAG® M2 Magnetic Beads (Sigma-Aldrich) according to the instructions provided by the manufacturer. In short, 1–2 mg of total protein lysate was incubated with 15 μl packed volume of anti-Flag M2 magnetic beads for 1 hour at room temperature and eluted at 98°C for 4 min in 2X Laemmli buffer (125 mM Tris-HCl pH 6.8, 20% glycerol, 4% SDS, 5% β-mercaptoethanol and 0.001% bromophenol blue), except for GFP-TRAP:GILT1, which was eluted in 0.1 M Glycine-HCl, pH 3.0.

#### Western blot

Immunoprecipitations and protein lysates were separated by SDS-PAGE and transferred onto a 0.2 μm PVDF membrane (Merck Millipore) using a semi-dry Trans-Blot Turbo transfer system (Bio-Rad). All membranes were blocked in 5% BSA in 1 x TBS (50 mM Tris-HCl, pH 7.5, 150 mM NaCl) for 1 hour at RT and incubated with primary antibodies overnight at 4°C. Antibodies were diluted in 1 x TBS with 5% BSA at the following dilutions; rabbit anti-Urm1 at 1:500 [29], mouse anti-Tubulin at 1:8000 (Sigma-Aldrich), mouse anti-Flag M2 at 1:1000 (Sigma-Aldrich), mouse anti-GFP JL-8 at 1:2000 (Clontech Laboratories/TaKaRa), mouse anti-HA antibodies at 1:1000 (Covance) and rabbit anti-Jafrac1 at 1:500 (kind gift from R. Lehmann [33]). The membranes were washed 5x for 10 min in TBST (1 x TBS with 0.075% Tween-20), incubated with HRP-linked secondary antibodies (GE Healthcare) for 1 h, and washed again 5 x 10 min, all at RT, before detection by ECL (Thermo Scientific or GE Healthcare) followed by autoradiography or C-Digit Blot Scanner analysis (LI-COR Biosciences). Protein size determination was established using either PageRuler prestained protein ladder (Thermo Scientific) or WesternSure prestained chemiluminescent protein ladder (LI-COR Biosciences). The complete protocol for Flag M2 immunoprecipitation and Western blot in *Drosophila* is available at dx.doi.org/10.17504/protocols.io.jivcke6 [PROTOCOLS DOI].

### Mass spectrometry and statistics analysis

The mass spectrometry (MS) analysis was performed together with the SciLifeLab facility for mass spectrometry based proteomics (Stockholm/Uppsala, Sweden), similar to [34] and as described below. The complete protocol for batch absorption of Urm1-Flag conjugates from *Drosophila* tissues for mass spectrometry analysis is available at dx.doi.org/10.17504/protocols.io.jizckf6 [PROTOCOLS DOI]. For each sample subjected to MS-based identification of Urm1-binding proteins, a batch-absorption of 9 mg total lysate, mixed with 75 μl packed volume of anti-Flag M2 magnetic beads (Sigma-Aldrich) was performed. The washed beads were stored in Lysis buffer A, supplemented with Complete Protease Inhibitor Cocktail (Roche), 1 mM PMSF and 10 mM N-Ethylmaleimide (Sigma-Aldrich), until they were subjected to on-bead trypsin digestion to allow identification of Urm1 associated proteins by MS. The resulting peptides were injected onto a LC-MS/MS system (UltimateTM 3000 RSLCnano chromatography system and Q Exactive Plus Orbitrap mass spectrometer, Thermo Scientific), where they were separated on a homemade C18 column, 25 cm (Silica Tip 360 μm OD, 75 μm ID, New Objective, Woburn, MA, USA) with a 60 min gradient at a flow rate of 300 nl/min. The gradient went from 5–26% of Buffer B (2% Acetonitrile, 0.1% Formic acid) in 120 min up to 95% of Buffer B in 5 min. The peptides obtained by the raw MS analysis was finally identified by screening the MS data against the Uniprot KB database.

The compiled MS data from the dual replicates of the control and Urm1-Flag rescued *Urm1*^*n123*^ samples were analyzed with the statistical R Studio software and visualised using the Gplots 3.1 package [35, 36]. Pairwise comparison of the rescue samples versus the control samples was performed using limma (moderated t-test). As relevant targets of urmylation, proteins that were either uniquely present in the *UAS*:*Urm1*^*3xFlag*^ rescued *Urm1*^*n123*^ samples, but not in controls, as well as proteins that were enriched to at least 3.5-fold higher levels in the rescue samples, as compared with the controls, were considered. The estimation of fold-change differences between the rescue versus control samples were based on the peptide signal intensity, together with the Benjamini-Hochberg corrected p-value, with an arbitrary cut-off of 1.3 and 0.3, respectively.

### Functional distribution analysis of the candidate Urm1 target proteins

Gene Ontology terms for the biological processes associated with each identified candidate Urm1 target protein were extracted from the FlyMine database [37] and subjected to statistical clustering using the DAVID (Database for Annotation, Visualization and Integrated Discovery) functional annotation clustering tool. The STRING database was employed to establish the association network of the complete set of newly identified Urm1-binding proteins [38]. Clustering of the gene ontology terms for the cellular component class was achieved using the PANTHER database [39].

## Results

In our previous research, we have identified the *Drosophila* homologues of Urm1 and Uba4 and provided evidence both for the occurrence of protein urmylation *in vivo* in fly tissues and a critical role for Urm1 in the response against oxidative stress [29]. *Drosophila* Urm1 shows high sequence similarity with both human (66% identity) and *S*. *cerevisiae* (37% identity) Urm1, contains the characteristic C-terminal GG motif of UBL proteins and display a predicted β-grasp fold [29]. During this initial work, we generated an Urm1 mutant (*Urm1*^*n123*^) in *Drosophila melanogaster*, in which the entire *Urm1* genomic locus has been deleted by imprecise excision of the *KG{SuPor-P}*^*KG08138*^ element [29]. A complete loss of Urm1 protein causes lethality in flies, but by reintroducing Urm1 under control of a ubiquitous GAL4 driver (Actin5C-GAL4), this lethality can be rescued [29]. In order to optimise the identification of Urm1 targets, we have thus generated a fly strain lacking endogenous Urm1, in which viability is supported by the ubiquitous expression of Flag-tagged Urm1 (*UAS*:*Urm1*^*3xFlag*^) under control of the Actin5C-GAL4 driver, expressed via the binary UAS/GAL4 expression system. Hence, all urmylation events that occur in this strain utilise the transgenically expressed Flag-Urm1 moiety and should be easily subjected to enrichment by affinity purification. Since the Flag-Urm1 rescued *Urm1*^*n123*^ flies display a restored viability and fertility [29], we consider the Flag-Urm1 protein to be suitable for the identification of biologically relevant targets of urmylation.

### Affinity purification of Urm1-associated proteins in *Drosophila melanogaster*

Having established a stable Flag-Urm1 rescued *Urm1*^*n123*^ fly strain, we set out to identify novel targets of urmylation at three distinctive stages of fly development; embryogenesis, late larval development (3^rd^ instar stage) and adulthood. The Flag-Urm1 rescued *Urm1*^*n123*^ fly strain and control flies were grown at 25°C to obtain a robust expression of Flag-Urm1. As control, we employed the progeny of *Actin5C-GAL4* females crossed to *w*^*1118*^ wild type *Drosophila* males, representing flies that lack Flag-Urm1 expression, but retain an unaltered endogenous Urm1 locus.

**Fig 1:**
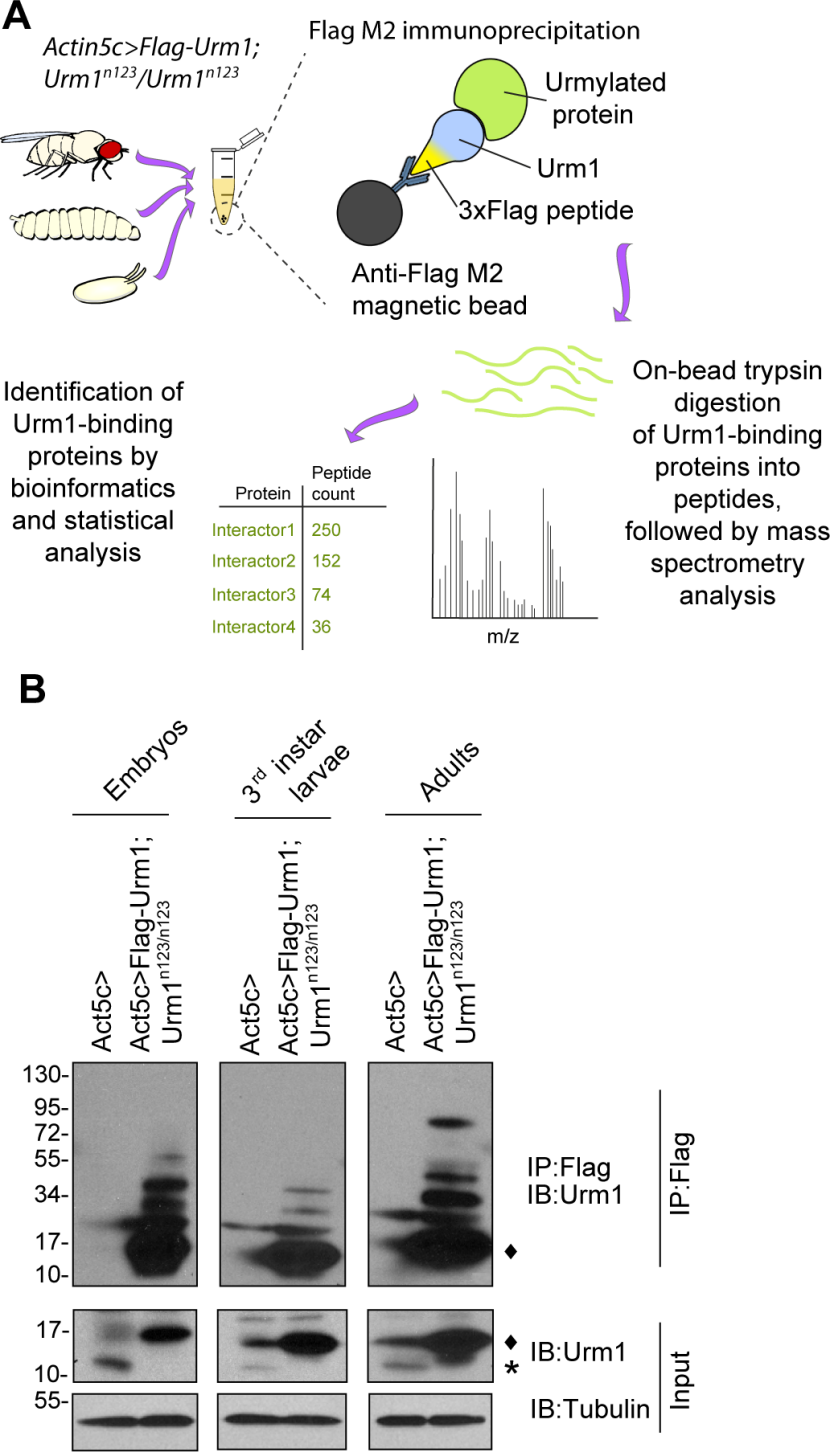
Strategy for identification of Urm1 conjugation targets in *Drosophila* embryos, larvae and adults *in vivo*. **A.** Schematic representation of the rationale for identifying Urm1-binding proteins and thereby candidate targets of urmylation during three critical stages of *Drosophila* development; embryogenesis, late larval stages and adulthood. Shortly, Flag-Urm1 associated proteins were enriched by immunoprecipitation with Flag M2 magnetic beads, which subsequently were subjected to on-bead trypsin digestion, followed by mass spectrometry and identification by standard bioinformatics analysis. **B.** Western blot illustrating the distribution of candidate Urm1 targets in embryos (left panel), larvae (middle panel) and adults (right panel), respectively. Urm1-interacting proteins were captured in the presence of NEM by Flag M2 immunoprecipitation, using magnetic beads, resolved under denaturing conditions by SDS-PAGE and detected by anti-Urm1 antibodies (* depicts endoge^◆^nous Urm1 expressed in control *Actin5C>w*^*1118*^ samples and indicates the unconjugated Flag-Urm1 fusion protein). Input represents 30 μg of the total lysate of the indicated genotypes.

For identification of Urm1 targets during embryogenesis, females of the Flag-Urm1 rescued *Urm1*^*n123*^ and the control strain were allowed to lay eggs on apple juice plates for 22 hours, to retrieve a collection of all embryonic stages. For the larval collection wandering L3 larvae were used, whereas adults were harvested 0–4 days after eclosure from the pupae. Protein lysates were prepared, in the presence of the isopeptidase inhibitor NEM to preserve urmylated proteins, for all three developmental stages and subjected to immunoprecipitation with Flag M2 magnetic beads to enrich for proteins associated with Flag-Urm1 (Fig 1A). By Western blot analysis, we could clearly observe an accumulation of multiple high molecular weight bands recognized by anti-Urm1 and anti-Flag antibodies specifically in flies that express Flag-Urm1, but not in the control lysates (Fig 1B and Panel A in S1 Fig). In agreement with representing proteins that interact with Flag-Urm1, these bands were abolished from the lysate after incubation with Flag M2 magnetic beads (Panel B in S1 Fig). Interestingly, the Urm1-positive bands showed diverging and distinctive patterns in embryos, larvae and adults, respectively, suggesting that Urm1 interacts with specific proteins during different biological processes.

### Identification of candidate *Drosophila* Urm1 targets by mass spectrometry

**Fig 2:**
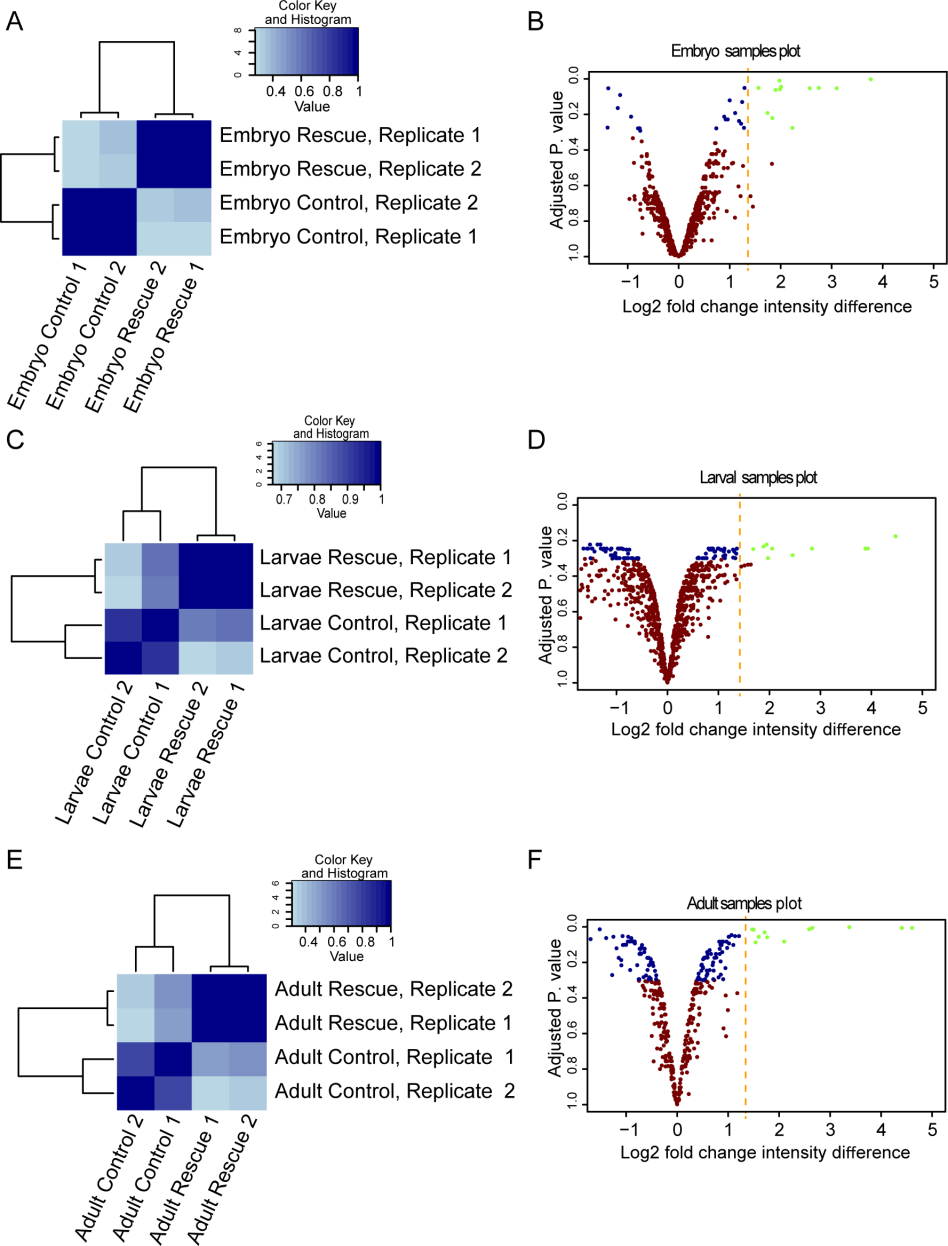
Identification of Urm1-binding proteins by mass spectrometry. **A, C, E.** Heat maps depicting a correlation analysis of the mass spectrometry results obtained from the two control *Actin5C>w*^*1118*^ replicate reads (Control replicate 1 and 2), versus the two Flag-Urm1 rescued *Urm1*^*n123*^ *Drosophila* replicate reads (Rescue replicate 1 and 2) for embryos (A), larvae (C) and adults (E), respectively. For all developmental stages, the two control and rescue replicates show high similarity, respectively, with a clear distinction between the controls versus the rescue samples. The color key code represents the Pearson correlation coefficient of the two replicates, where 1 depicts 100% similarity between the two replicates and 0 depicts no correlation between the samples. **B, D, F.** Volcano plots illustrating the magnitude of differential distribution (log2 fold-change) of the signal intensity between the Flag-Urm1 rescue and the control samples, together with the adjusted p-value for embryonic (B), larval (D) and adult (F) samples, respectively. Red dots depict peptides that displayed a log2 fold-change of less than 1.3 and a high adjusted p-value, when comparing the rescue and control samples. Blue dots represent peptides that did not show any significant difference between the rescue and the control samples, with a lower adjusted p-value. Green dots pinpoint peptides that demonstrated a low adjusted p-value and were enriched in the rescue samples to a minimum of 1.3 log2 fold-change, as compared with the control samples. The green dots represent the peptides that were finally singled out as Urm1-interacting proteins, with the cut-off marked by a dashed yellow line.

To uncover the identity of the candidate targets of urmylation, the Flag M2 magnetic beads were subjected to on-bead trypsin digestion followed by analysis by mass spectrometry [34] (Fig 1A). The analysis was performed using two biological replicates for the embryonic, larval and adult samples, respectively. Within each developmental stage, there was a high correlation between the identified peptides as well as their signal intensity, when comparing the two replicates (Fig 2A, 2C and 2E, together with S2 Fig). This ascertains a high confidence for pinpointing biologically relevant Urm1-binding proteins and putative targets of urmylation in these samples.

When continuing the identification of putative Urm1 targets, we set up two criteria for selecting relevant candidates. As top candidates to be modified by urmylation, we first singled out proteins that were detected in the Flag-Urm1 rescue samples, but not in the controls. Secondly, these primary hits were complemented by proteins that were present in both the rescue and control samples, but that displayed at least a 3.5-fold increase in the Flag-Urm1 rescued animals, as compared with the controls (Fig 2B, 2D and 2F). Based on these criteria, we retrieved a list of 79 unique Urm1-associated proteins and thus putative targets of Urm1 conjugation with either exclusive or overlapping expression patterns, including 35 proteins during embryogenesis, 30 proteins in late larval stages and 30 proteins in adult flies (Table 1).

**Table 1:** Compiled list of the Urm1-interacting proteins in *Drosophila* embryos, larvae and adults, identified by mass spectrometry.

CG number	Gene	CG number	Gene
Identified in embryos, larvae and adults		Identified in larvae	
CG13090	Uba4	CG10130	Sec61beta
CG12013	PHGPx	CG11241	CG11241
CG7217	Prx5	CG11893	CG11893
CG7266	MsrA/Eip71CD	CG12908	Ndg
CG8078	CG8078	CG1365	CecA1
CG9796	GILT1	CG14500	CG14500
**Identified in** **embryos and adults**		CG15877	CG15877
		CG2233	CG2233
CG1633	Jafrac1	CG31974	CG31974
CG3989	ade5	CG33095	CG33095
**Identified in** **larvae and adults**		CG3401	betaTub60D
		CG4528	snf
CG12279	CG12279	CG4679	CG4679
CG7597	Cdk12	CG5516	CG5516
**Identified in embryos**		CG6174	Arp1
		CG6340	CG6340
CG10189	CG10189	CG7516	l(2)34Fd
CG10918	CG10918	CG8031	CG8031
CG11208	CG11208	CG8709	Lpin
CG11739	CG11739	CG9577	CG9577
CG12262	CG12262	CG9586	CG9586
CG12740	RpL28	CG9633	RpA-70
CG12797	Ciao1	**Identified in adults**	
CG13630	CG13630		
CG1441	CG1441	CG10460	cer
CG15481	Ski6	CG10992	CtsB1
CG1710	Hcf	CG11765	Prx2540-2
CG1782	Uba1	CG12405	Prx2540-1
CG1972	IntS11	CG15261	UK114
CG2151	Trxr-1	CG15697	RpS30
CG32346	E(bx)	CG1594	hop
CG3564	CHOp24	CG16757	Spn
CG3931	Rrp4	CG17051	dod
CG41128	CG41128	CG1721	Pglym78
CG4463	Hsp23	CG17320	ScpX
CG5933	Ime4	CG2559	Lsp1alpha
CG6523	CG6523	CG2843	Cwc25
CG6677	ash2	CG31793	CG31793
CG7010	l(1)G0334	CG3717	bcn92
CG8479	Opa1	CG4178	Lsp1beta
CG8983	ERp60	CG5502	RpL4
CG9128	Sac1	CG6821	Lsp1gamma
CG9911	CG9911	CG6871	Cat
		CG8201	par-1

### Characterisation of putative Urm1 targets in *Drosophila melanogaster*

**Fig 3:**
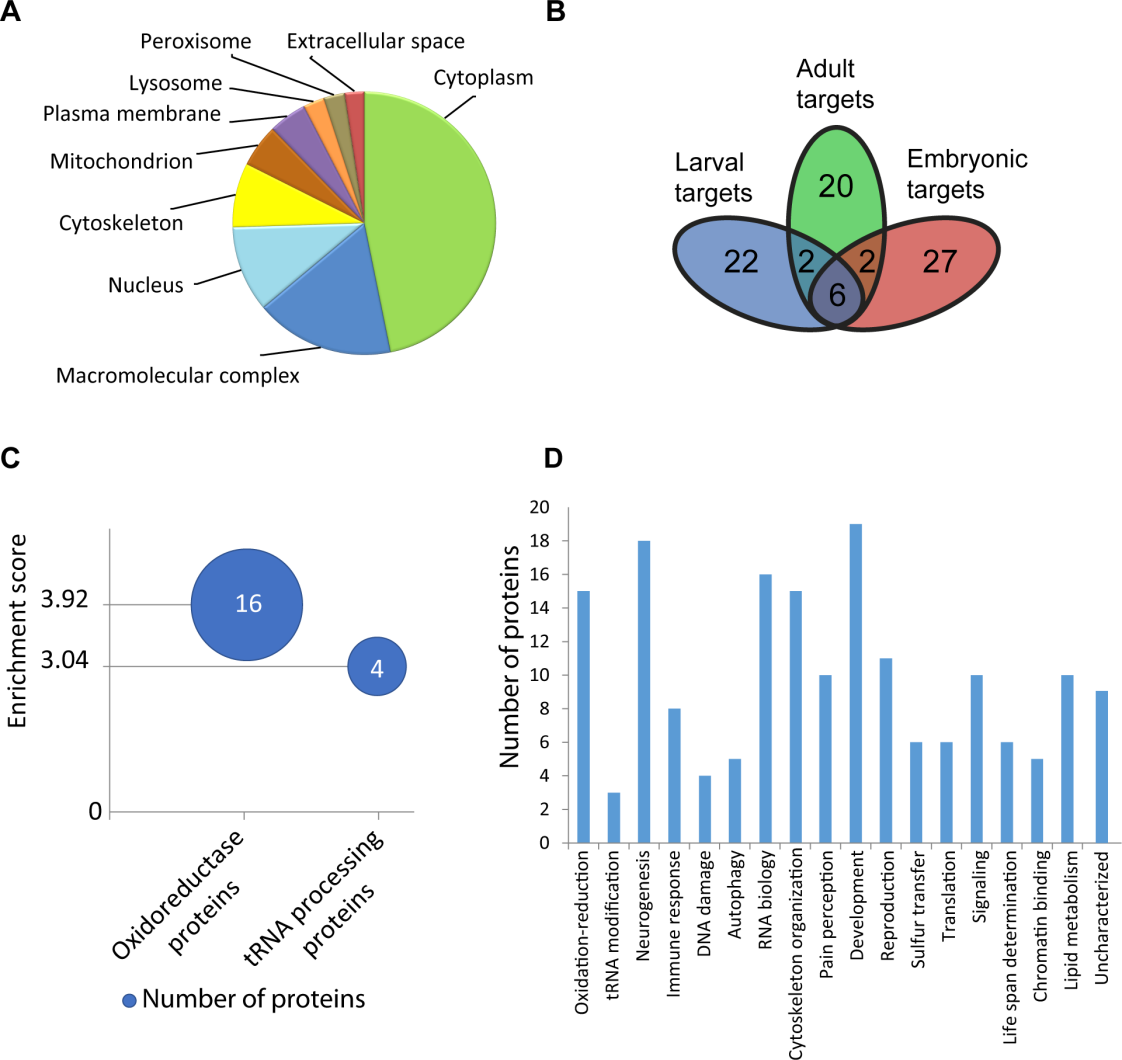
Functional characterization of the newly identified candidate targets of Urm1 conjugation in *Drosophila*. **A.** Subcellular distribution of the proteins identified as Urm1-binding partners by mass spectrometry, depicting an accumulation of candidate targets of urmylation in the cytoplasmic compartment and/or associated with distinct membrane-bound organelles. **B.** Venn diagram illustrating the amount of unique versus shared Urm1-interacting proteins in the developmental stages investigated; embryos, larvae and adults. **C.** Functional clustering of the Urm1-associated proteins established using the DAVID database, suggesting that Urm1 most likely displays its most important functions in oxidation-reduction processes and tRNA modification. Enrichment scores of >3.0 were considered as meaningful. **D.** A functional classification of the Urm1-binding proteins based on gene ontology (GO) classification suggests that Urm1 may be involved in multiple different biological processes.

Previous studies, including our own initial characterisation of Urm1 in *Drosophila* [29], have depicted Urm1 as a protein primarily residing in the cytoplasmic compartment. Consistent with these findings, the majority of the Urm1 binding partners identified in this study are proteins known to localise in the cytosol, either unbound or associated with macromolecular complexes, cytoskeletal structures or membrane-bound compartments such as peroxisomes or lysosomes (Fig 3A). Interestingly 10% of the identified Urm1 associated proteins are classified as nuclear, which suggests that Urm1 may also display yet uncharacterized functions in the nucleus. Based on functional designation of these nuclear candidate targets, Urm1 may thus be involved in processes such as transcriptional regulation and chromatin remodelling, as well as DNA damage checkpoints and responses (S3 Fig).

Among the in total 79 unique proteins that were identified as candidate Urm1 targets in *Drosophila*, the majority was exclusively found in either the embryonic, larval or adult preparations (Fig 3B). Only six (~8%) of the candidates occurred at all stages investigated, namely the Urm1 E1 activating enzyme Uba4, the fly homologue of the tRNA 2-thiolation protein Ncs6 (*CG8078*), Gamma-interferon-inducible lysosomal thiol reductase 1 (GILT1), the oxidation-reduction associated proteins Phospholipid Hydroperoxide gluthathione peroxidase (PHGPx) and Peroxiredoxin 5 (Prx5), as well as Methionine sulfoxide reductase A (MsrA), also known as Ecdysone-induced protein 28/29kD (Eip71CD). The identification of Uba4 and Prx5 as *Drosophila* Urm1-binding partners by mass spectrometry strongly emphasizes the relevance of capturing candidate Urm1 targets with Flag-Urm1, since a solid body of evidence has already established an evolutionary conserved interaction of Urm1 with both Uba4 and Ahp1/Prx5 in yeast, *Drosophila* and mammalian cells [5, 17, 19, 29, 30]. Among the rest, the peroxiredoxin Jafrac1 and the glycinamide ribotide synthetase and aminoimidazole ribotide synthetase Ade5 were commonly identified as Urm1 binding partners in embryos and adults, whereas Cyclin-dependent kinase 12 (Cdk12) and the predicted thiosulphate sulfurtransferase gene *CG12279* were detected both in larvae and adults. The remaining 69 candidate target proteins were consequently found solely in embryos, larvae or adults.

However, despite this diversity in targets when comparing the different developmental stages investigated, functional analysis of these proteins indicate a clustering of Urm1 involvement in specific biological processes. In agreement with the to date published information on Urm1, functional clustering of the Urm1-associated proteins identified in this study indicates the most critical Urm1-regulated activities to be oxidation-reduction processes and tRNA modification (Fig 3C). A compilation of the gene ontology terms associated with the newly identified Urm1-associated partners additionally suggests that Urm1 may also display functions in immune responses, autophagy, DNA damage control, pain perception, mRNA processing and translation, as well as reproduction and life span determination (Fig 3D and S3 Fig). Indeed, a putative role of Urm1 in reproduction and life span determination is completely in line with the reduced fertility and shortened life span displayed by flies deficient of Urm1 expression [29]. Moreover, the Urm1 candidate targets may also point toward an association of Urm1 with multiple intracellular signalling pathways, in addition to several critical developmental events, since many of the identified binding partners have established functions both in early embryogenic processes such as axis determination and dorsal closure, as well as the development of specific organ systems, including the nervous system, heart, trachea and somatic musculature (Fig 3D).

**Fig 4:**
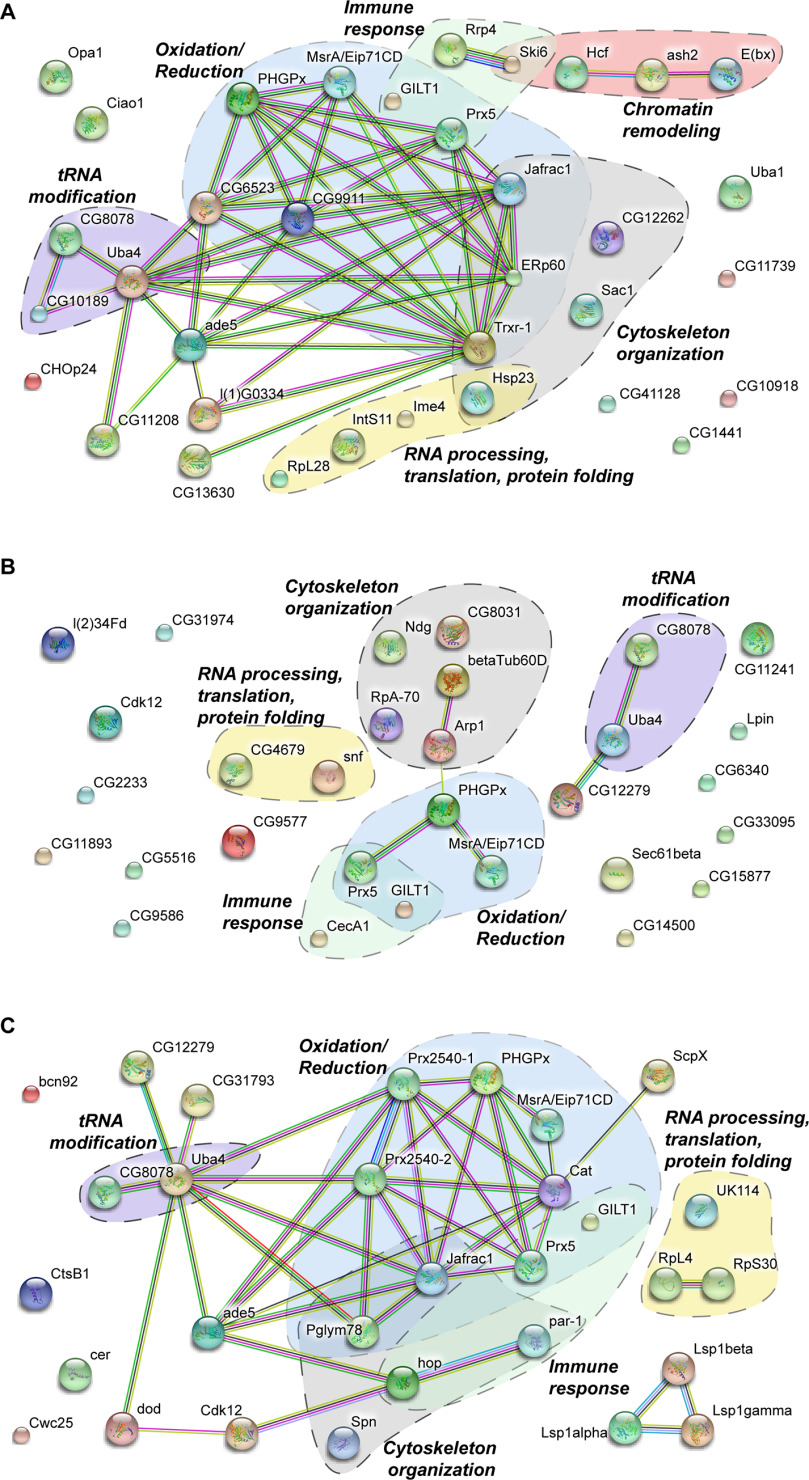
Protein-protein interaction analysis by STRING clusters Urm1 conjugation targets in multiple distinctive functional networks. STRING analysis of the Urm1-intercting partners identified by mass spectrometry, depicting functional networks where Urm1 may be involved in embryos (A), larvae (B) and adult flies (C). During all developmental stages, Urm1 appears to be associated with networks of proteins that regulate oxidation-reduction processes, tRNA modification, immune responses, mRNA processing, translation and protein folding, as well as cytoskeletal dynamics. In embryos, Urm1 is additionally linked to a protein network which is involved in chromatin remodeling.

This emerging pattern of a dynamic landscape of Urm1 activity is further reinforced when employing the STRING database to generate a protein-protein association network analysis of the newly identified Urm1 target proteins from all developmental stages (Fig 4). In agreement with the functional clustering, STRING analysis exposes extensive protein networks associated with Urm1 in tRNA modification and oxidation-reduction activities, but further also positions Urm1 in networks regulating mRNA processing, translation and protein folding, as well as immune responses, chromatin remodelling and cytoskeletal dynamics. Whereas chromatin remodelling proteins appears to be linked to Urm1 uniquely during embryogenesis (Fig 4A), Urm1-interaction partners from all the other major functional clusters are present at all developmental stages, including embryos (Fig 4A), larvae (Fig 4B) and adult flies (Fig 4C).

### Confirmation of candidate Urm1 target proteins

**Fig 5:**
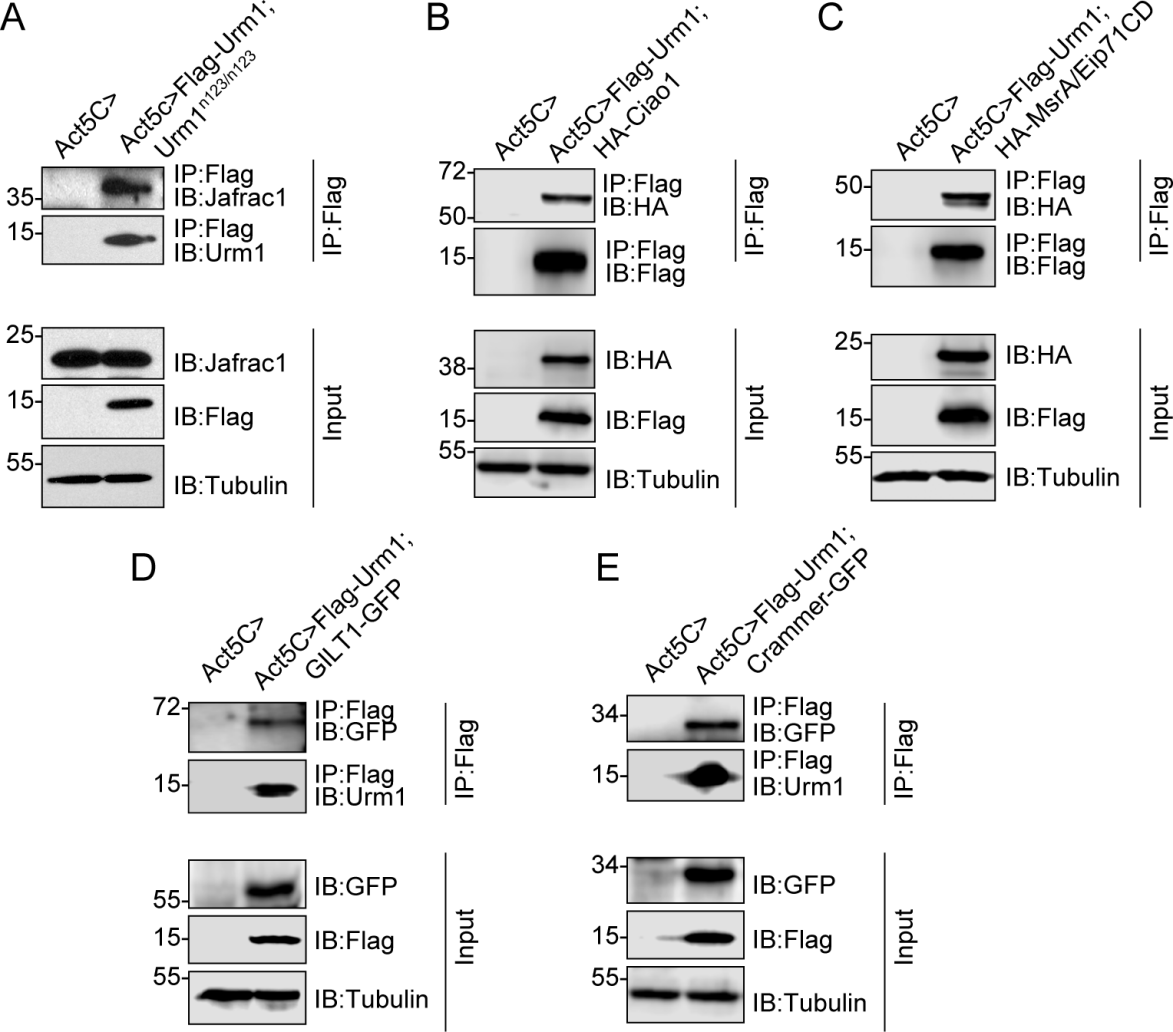
*In vivo* confirmation of the binding of Urm1 to a panel of target proteins identified by mass spectrometry. Confirmation of a physical interaction between Urm1 and five of the newly identified candidate targets of urmylation. Using the UAS/GAL4-system, Flag-Urm1 was either expressed alone, or co-expressed separately with HA-Ciao1, HA-MsrA/Eip71CD, GFP-GILT1 or GFP-Crammer, respectively, under control of the Actin5C-GAL5 driver. In protein lysates from the resulting flies, the interaction between Urm1 and the candidate target proteins were subsequently analyzed by immunoprecipitation, performed in in the presence of NEM. By immunoblotting Flag-Urm1 immunoprecipitates with anti-Jafrac1, anti-HA or anti-GFP antibodies, an interaction could thereby be verified between Flag-Urm1 and endogenous *Drosophila* Jafrac1 (A), HA-tagged Ciao1 (B), HA-tagged MsrA/Eip71CD (C), GFP-tagged GILT1 (D) and GFP-tagged Crammer (E). When comparing the molecular weights of the candidate Urm1 target proteins following immunoprecipitation, a size shift of ~15 kDa could be observed for Jafrac1 (A), HA-Ciao1 (B), HA- MsrA/Eip71CD (C) and GFP-GILT1 (D) as compared with protein lysate controls, which is in agreement with the covalent conjugation of one Flag-Urm1 moiety (e.g. target protein urmylation). In contrast, GFP-Crammer displayed the same molecular weight in both Flag-Urm1 immunoprecipitates and crude fly lysates, depicting a non-covalent mode of interaction, which is sensitive to denaturing conditions (E). Input represents 30 μg of the total lysate of the indicated genotypes.

In order to validate our strategy of identifying putative targets of Urm1 conjugation by mass spectrometry, we wished to confirm the urmylation of a panel of candidate proteins *in vivo*. Importantly, in our previous studies we have already verified an interaction between *Drosophila* Urm1 and Uba4, as well as Prx5 [29]. By screening the available public collections of *Drosophila* fly strains, including the Bloomington *Drosophila* Stock Center, FlyORF and Kyoto Stock Center (DGRC), as well as the current literature, we managed to obtain tools suitable for addressing the urmylation status of several of the identified targets by immunoprecipitation. Firstly, we identified the peroxiredoxin Jafrac1 as a target of urmylation, since Jafrac1 could be clearly observed in immunoprecipitates together with Flag-Urm1, detected by custom-made antibodies against Jafrac1 [33]. In agreement with a covalent conjugation between these two proteins, Jafrac1 displayed a size shift of ~15 kDa upon co-immunoprecipitation with Flag-Urm1 under denaturing and reducing conditions, as compared with endogenous Jafrac1 in fly lysates, corresponding to the addition of one Flag-Urm1 moiety (Fig 5A). Secondly, we co-expressed HA- or GFP-tagged fusion variants of the candidate target proteins Ciao1 (Fig 5B), MsrA/Eip71CD (Fig 5C) and GILT1 (Fig 5D) together with Flag-Urm1 in *Drosophila*, and could similar to Jafrac1 observe an interaction between these proteins and Flag-Urm1, accompanied by a ~15 kDa size shift of the target proteins. Interestingly, when aiming at also verifying Crammer as a target of urmylation by analysing the interaction between Flag-Urm1 and GFP-tagged Crammer, we found that the proteins indeed interacted in fly lysates, but without the characteristic size shift in Crammer (Fig 5E). This lead us to the conclusion that rather than being urmylated, GFP-Crammer instead appears to interact with Urm1 by means sensitive to denaturing conditions, thus depicting a non-covalent mechanism of interaction (Fig 5E). Taken together with our previous work [29], we can thus conclude that among the 79 Urm1-binding partners identified in this study by mass spectrometry, we have confirmed a physical interaction between Urm1 and seven of these proteins.

## Discussion

In this report, we take the knowledge of Urm1-modified target proteins from the initial analysis performed in yeast and mammalian cells [17, 19] to the next level, by identifying 79 Urm1-interacting proteins in *Drosophila melanogaster*. This first undertaking to define urmylated proteins in a multicellular organism puts a new focus on this poorly characterised UBL modifier and contributes with several important pieces of information to enhance the understanding of the physiological role of Urm1. In our previous work we have found the urmylation landscape to be highly dynamic, with distinctive patterns of Urm1 conjugates in different developmental stages [29]. Hence, to identify as many Urm1 targets as possible and expose different biological functions of urmylation, we chose to target three key stages of *Drosophila* development for our analysis, namely embryogenesis, larval development and adulthood. Using this strategy, we could conclude that as suspected only a handful of the 79 identified candidate targets were shared throughout development, whereas most proteins appeared to be uniquely associated with Urm1 at a particular time in development. Importantly, as confirmed by ourselves before [29], and in agreement with data from *S*. *cerevisiae* and mammalian cells [5, 19, 30], Urm1 was found in complex with its E1 activating enzyme Uba4 in all stages examined. In order to verify the suitability of our methodology for identifying candidate Urm1 targets of biological relevance, we further continued our investigations by confirming a physical interaction between Urm1 and five of the candidate target proteins. Among these, four was found to interact with Urm1 in samples denatured by SDS and reduced in the presence of β-mercaptoethanol, in a manner that resulted in a size shift of the target protein corresponding to the addition of one Flag-Urm1 moiety, thus indicating a covalent conjugation to Urm1.

Similar to the findings in yeast and mammalian cells, our results suggest that *Drosophila* Urm1 most likely displays its most important functions during oxidative stress and tRNA modification. A conservation of the most well-characterized role of Urm1 as a sulfur carrier in the 2-thiolation of the wobble uridine (U_34_) of certain cytosolic tRNAs [6–10], seem to also be relevant in flies, since the *Drosophila* homologues of two key proteins involved in this process, *Nsc2/ATPBD3* (*CG10189*) and *Ncs6/C16ORF84* (*CG8078*), were found among the Urm1-binding partners. In keeping with this conclusion, NCS6 and NCS2 (also known as CTU1/ATPB3 and CTU2, respectively) have previously also been pinpointed as targets of urmylation in mammalian cells [19].

In contrast, there are several pieces of evidence that argue for a key role of Urm1 in oxidation/reduction processes in flies. This conclusion is supported both by the increased resistance to oxidative stress displayed by *Drosophila Urm1*^*n123*^ null mutants, and further reinforced by the accumulation of proteins involved in oxidation-reduction processes among the newly identified Urm1-interacting proteins. In addition to Prx5 [29], we have now also confirmed a second peroxiredoxin, Jafrac1, as well as MsrA/Eip71CD and GILT1 as targets of Urm1 conjugation in flies, all strongly associated with the response against oxidative stress [40–42]. The establishment of MsrA/Eip71CD urmylation in flies is further in line with the identification of MsrA as a target of SAMPylation in *H*. *volcanii* [24], suggesting that MsrA/Eip71CD is an evolutionary conserved target of Urm1 conjugation. Moreover, despite the lack of evidence for an involvement of Sac1 in oxidative stress, the identification of Sac1 as a candidate target of urmylation is highly interesting in this aspect, in light of its reported role as a repressor of JNK pathway activation in flies [43]. *Urm1*^*n123*^ mutants display a marked increase in JNK signalling, including JNK-regulated gene transcription of *Jafrac1* and *gstD1* [29], and possibly Sac1 could be the missing link in this genetic framework.

The modification of target proteins by UBL modifiers often occur in response to some type of external stimuli or cellular stress, such as DNA damage or stimulation of the immune system [44, 45]. When functionally clustering the Urm1-associated proteins in *Drosophila*, it is obvious that this also holds true for fly Urm1. In addition to the established role in oxidative stress, the Urm1 candidate targets pinpoint an involvement also in the response against DNA damage and immunological threats, as well as stressful stimulation of the nervous system in the form of pain. Since reactive oxygen species (ROS) are commonly utilised as signalling molecules during viral infections, it is not surprising that genetic networks that regulate oxidation-reduction biology also has an impact on immune functions [46]. Dual functions during oxidative and immunological stress has indeed been reported for both Prx5 [47, 48] and GILT1 [40, 49] in *Drosophila*, two of the confirmed targets of urmylation. Similarly, there is a strong connection between immune responses, oxidative stress and autophagy [50]. In this context it is worth noting that five of the newly identified Urm1-associated proteins also display documented roles in autophagy, including Cecropin A1 (CecA1), Cathepsin B1 (Cts B1) and Uba1.

Even though urmylation primarily seems to occur in the cytoplasmic compartment or in the vicinity of membrane-bound organelles, it is interesting to note that also nuclear Urm1-binding partners are prevalent, including chromatin-associated proteins involved in DNA damage control and the mitotic G2 checkpoint, as well as regulators of chromatin organization and transcription. One of these that are of particular interest is Ash2, a shared component of the three COMPASS (complex associated with Set1) complexes in *Drosophila*, which all induce Histone H3 Lysine 4 (H3K4) methylation of DNA, thus labelling euchromatin and regions of active transcription [51]. In one of these complexes, MLL1/MLL2 (mixed lineage leukemia), Ash2 interacts with the MLL protein and homeotic gene *Trithorax (Trx)*, which regulates the expression of Hox genes. Hox genes are master regulators of development and cell fate specification [51, 52], which is striking in light of the early embryonic lethality displayed by *Urm1*^*n123*^ mutant embryos that lack maternal contribution of Urm1. A possible involvement of Urm1 in the regulation of transcription by the COMPASS complexes is reinforced also by the identification of another MLL-associated protein, the Herpes simplex virus host cell factor Hcf, as a putative target of urmylation, an interactor of both Polycomb Group (PcG) and Trithorax Group (TxG) genes [53]. The detection of Urm1 binding partners such as Ski6, associated with transcriptionally active chromatin [54] and Enhancer or bithorax (E(bx)), involved in the regulation of transcription [55–57], again positions Urm1 in protein networks that regulate gene expression. Target proteins identified in this study, as well as in human cells [19], in addition point towards a role of Urm1 also later in the gene expression process, since these include proteins involved in mRNA binding and processing, translation and protein folding.

The early embryonic lethality in *Urm1*^*n123*^ mutants lacking maternally contributed Urm1 could alternatively also be associated with several of the other Urm1-associated proteins, such as Par-1, which is an evolutionary conserved master regulator of cell polarity and axis determination [58]. In light of our previously described role of Urm1 in JNK activation, the position of Par-1 at the intersection between JNK and Wnt signalling during development [59] may suggest that the involvement of Urm1 in different biological processes could be mediated by targeting the JNK pathway in different tissues and distinct developmental time points. Besides Par-1, the embryonically lethal genetic locus *Inducer of Meiosis 4 (Ime4)* is another highly interesting hit among the new candidates of urmylation. In addition to embryonic lethality, loss of *Ime4* in *Drosophila* results in reduced fertility and shortened lifespan [60], thus phenocopying three key characteristics of *Urm1*^*n123*^ null mutants. The reduced longevity caused by loss of *Urm1*, which is prominent among adult zygotic homozygous *Urm1*^*n123*^ mutant escapers, can moreover also be correlated with similar phenotypes that has been reported for several of the newly identified Urm1-associated proteins, such as Prx5 [48], Jafrac1 [41], MsrA/Eip71CD [61] and Trxr-1 [62].

In our endeavour to verify at least a handful of the candidate Urm1-interacting proteins as *bona fide* targets of urmylation, we could besides observing a covalent conjugation of Urm1 to multiple target proteins also detect one Urm1-associated protein that appeared to interact with Urm1 by non-covalent means, Crammer. It is intriguing to speculate whether Crammer may contain a protein domain or motif that specifically binds to Urm1. Protein domains assigned for recognizing urmylated proteins have to date not been described, but would not be surprising, as specialised domains for the recognition of posttranslational modifications are common [63].

Taken together, in this report we have identified 79 proteins that interact with the ubiquitin-like protein Urm1 in *Drosophila melanogaster*, either by covalent conjugation (e.g. urmylation) or non-covalent protein-protein interaction. Among these 79 proteins, most are completely novel and pinpoint multiple cellular processes in which Urm1 may have a function that is yet to be explored. Besides confirming the importance of Urm1 in tRNA modification, the identities of the new candidate targets of urmylation place Urm1 at the intersection of multiple biological pathways that in several cases are related to cellular stress, general health and longevity. The results of this study may now serve as a starting point for addressing many questions regarding the function of Urm1 in various biological processes, mapping of putative urmylation motifs and Urm1-binding domains, and eventually unearthing the importance of urmylation in a multicellular organism.

## Supporting information

S1 Fig Differential distribution of Urm1-conjugated proteins in different developmental stages. **A.** Western blot illustrating the presence and distribution of Flag-Urm1 conjugated proteins in total fly lysates of control *Act5C>* and *Act5C>*^*3xFlag*^*Urm1; Urm1*^*n123/n123*^ embryos, larvae and adults, respectively. The image depicts a unique urmylation pattern in different developmental contexts, as recognized by anti-Flag antibodies.
**B.** Following incubation with Flag M2 magnetic beads, the high molecular weight bands recognized by anti-Urm1 antibodies in Flag-Urm1 rescued *Urm1*^*n123*^ adult flies are abolished from the lysate, indicating that these bands represent proteins that interact with Urm1. Western blot analysis of protein lysates from either control *Act5C>* or *Act5C>*^*3xFlag*^*Urm1; Urm1*^*n123/n123*^ flies, before and after incubation with Flag M2 magnetic beads.
(TIF)

S2 Fig A high correlation between the analysed replicates indicates a high confidence for the identification of novel Urm1-associated proteins. Scatter plots demonstrating the Pearson correlation between the two individual replicates of *Actin5C>w*^*1118*^ control and *Act5C>*^*3xFlag*^*Urm1; Urm1*^*n123/n123*^ rescue samples for embryos (A), larvae (B) and adults (C), respectively.
(TIF)

S3 Fig Stage-specific presentation of the biological processes that are associated with the newly identified Urm1-interacting proteins in *Drosophila*. Detailed analysis of the biological processes with which the newly identified Urm1-interacting proteins are associated in embryos (A), larvae (B) and adults (C), respectively. The analysis is based on the gene ontology terms linked to each individual Urm1-interacting protein. Biological processes associated with two or more proteins are clustered in the pie chart, whereas the remaining fall into the “other” category.
(TIF)
