# Supplementary figures and images for: A proteomics approach to identify targets of the ubiquitin-like molecule Urm1 in Drosophila melanogaster
Source: PLoS One. 2017 Sep 27;12(9):e0185611. doi: 10.1371/journal.pone.0185611 (PMC5617222; doi:10.1371/journal.pone.0185611)

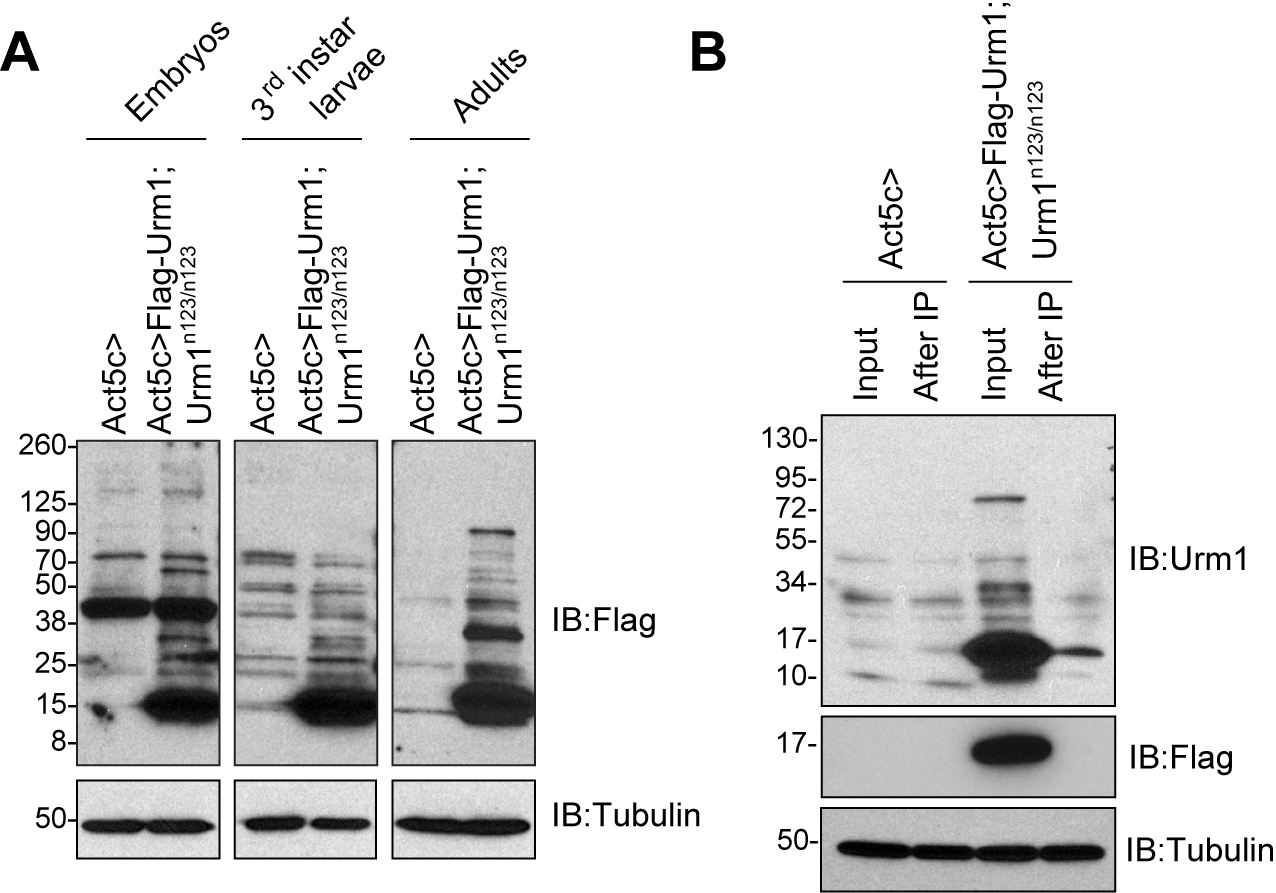

Supplement: S1 Fig — A. Western blot illustrating the presence and distribution of Flag-Urm1 conjugated proteins in total fly lysates of control Act5C> and Act5C>3xFlagUrm1; Urm1n123/n123 embryos, larvae and adults, respectively. The image depicts a unique urmylation pattern in different developmental contexts, as recognized by anti-Flag antibodies. B. Following incubation with Flag M2 magnetic beads, the high molecular weight bands recognized by anti-Urm1 antibodies in Flag-Urm1 rescued Urm1n123 adult flies are abolished from the lysate, indicating that these bands represent proteins that interact with Urm1. Western blot analysis of protein lysates from either control Act5C> or Act5C>3xFlagUrm1; Urm1n123/n123 flies, before and after incubation with Flag M2 magnetic beads. (TIF) [file pone.0185611.s001.tif]

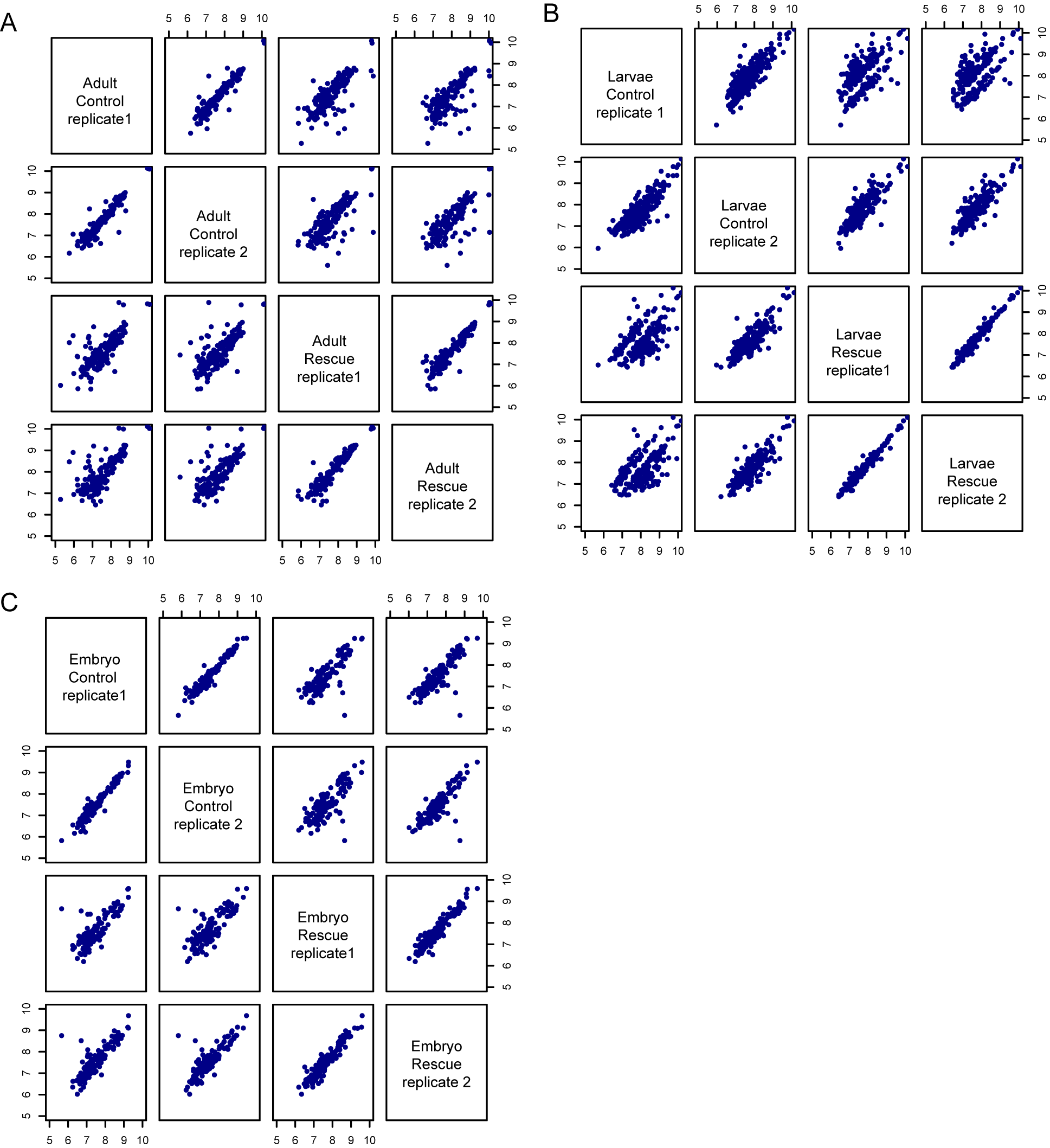

Supplement: S2 Fig — Scatter plots demonstrating the Pearson correlation between the two individual replicates of Actin5C>w1118 control and Act5C>3xFlagUrm1; Urm1n123/n123 rescue samples for embryos (A), larvae (B) and adults (C), respectively. (TIF) [file pone.0185611.s002.tif]

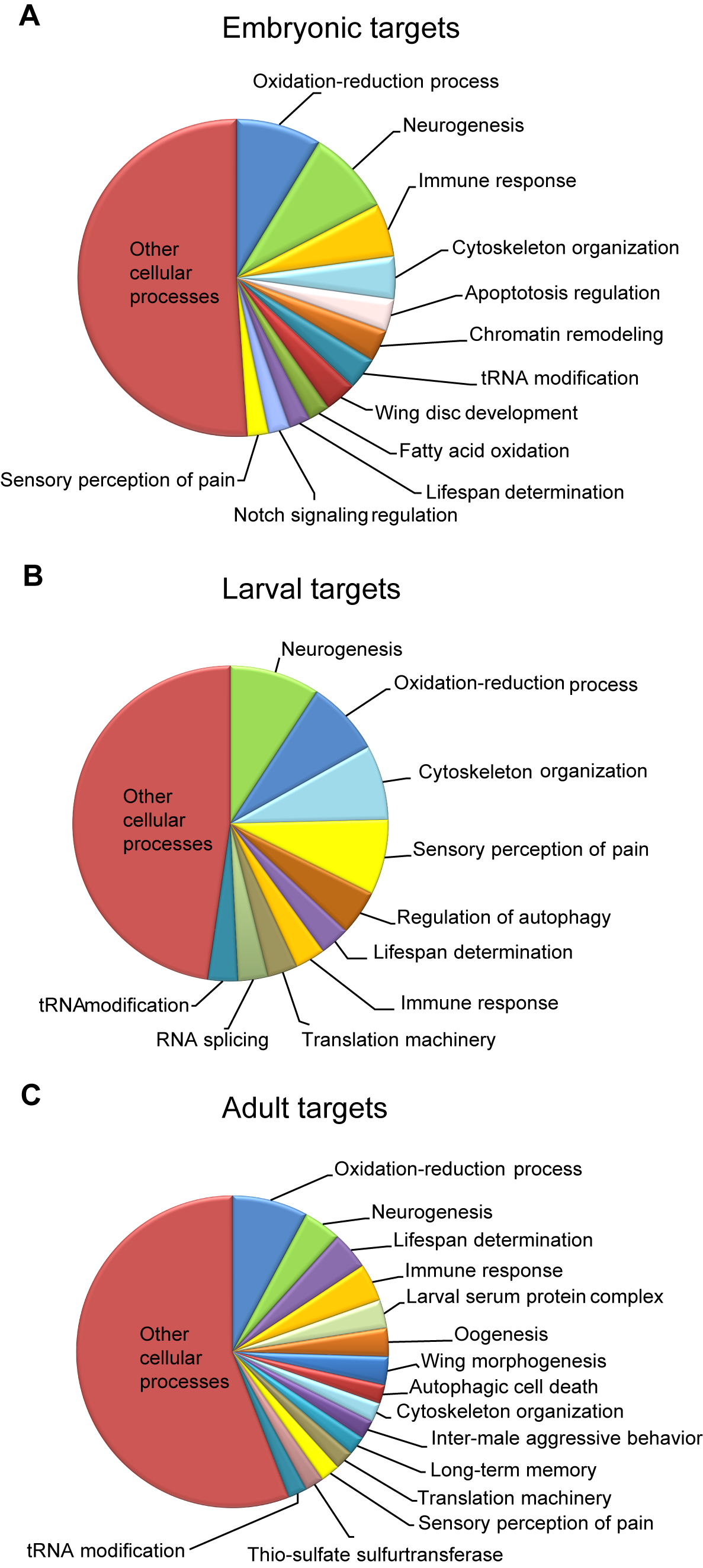

Supplement: S3 Fig — Detailed analysis of the biological processes with which the newly identified Urm1-interacting proteins are associated in embryos (A), larvae (B) and adults (C), respectively. The analysis is based on the gene ontology terms linked to each individual Urm1-interacting protein. Biological processes associated with two or more proteins are clustered in the pie chart, whereas the remaining fall into the “other” category. (TIF) [file pone.0185611.s003.tif]
